# Radon and childhood cancer

**DOI:** 10.1038/sj.bjc.6600671

**Published:** 2002-11-12

**Authors:** D L Henshaw

**Affiliations:** HH Wills Physics Laboratory, University of Bristol, Tyndall Avenue, Bristol BS8 1TL, UK

## Abstract

*British Journal of Cancer* (2002) **87**, 1336–1337. doi:10.1038/sj.bjc.6600671 www.bjcancer.com

© 2002 Cancer Research UK

## 

**Sir**

The recent United Kingdom Childhood Cancer Study of exposure to domestic sources of ionising radiation: 1: radon gas (UKCCS Investigators, *British Journal of Cancer* (2002) **86:** 1721–1726) found no evidence of increased risk in relation to exposure to domestic sources of radon, but the authors found evidence that their control houses tended to have intrinsic features resulting in higher than average indoor radon concentrations.

As the UKCCS Investigators point out, radiation risk estimates suggest that approximately 14% of the incidence of childhood leukaemia in the UK may be linked to natural background high-LET alpha-radiation (Committee on Medical Aspects of Radiation in the Environment, 1996; Simmonds *et al*, 1995). Partitioning the dose to red bone marrow between the principal emitters suggests that 5% of childhood leukaemia may be linked to ^222^Rn, ^220^Rn and their short-lived alpha-emitting decay products, and 9% to longer-lived emitters, especially ^210^Po. This suggests that for radon gas concentrations of 20, 100 and 200 Bq m^−3^, the relative risks of childhood leukaemia with respect to zero radon are respectively 1.05, 1.25 and 1.50. These values also define the resolving power of a case–control study to detect this level of risk at a given radon concentration.

Such resolving power has not been available in any previous case–control study, including those cited by the UKCCS Investigators (Stjernfeldt *et al*, 1987; Lubin *et al*, 1998; Kaletsch *et al*, 1999; Steinbuch *et al*, 1999). Even without the biases in the controls identified by the UKCCS Investigators, this was also true of their study as there were too few cases in the high radon exposure category.

However, I suggest that there are at least three other factors which limit the ability to detect a link between radon and childhood cancer in the United Kingdom Childhood Cancer Case–control Study and which pose serious challenges for the design of future studies.

Firstly, we have shown that the level of ^210^Po in children's teeth, a marker for the level in the skeleton, varies considerably between children (Henshaw *et al*, 1994), in particular higher levels are associated with proximity to major sources of vehicle exhaust pollution in the UK (Henshaw *et al*, 1995). Examination of our database suggests that for children living in rural areas, the average activity concentration of ^210^Po in permanent teeth extracted for orthodontic purposes is approximately 7.2 Bq kg^−1^. Near motorways the average activity concentration is approximately 10.6 Bq kg^−1^, but the range can extend up to 18 Bq kg^−1^ and occasionally even higher concentrations have been recorded. Using the corresponding bone marrow dose estimates given in Simmonds *et al* (1995), the possible variations in ^210^Po skeletal burden correspond to an equivalent spread in radon exposure of about 90 Bq m^−3^. In the UKCCS, the level of ^210^Po in the skeleton of case and control children is not known, nevertheless there is potential for serious confounding of total bone marrow dose from high-LET emitters, given that the highest radon bin is only 200+ Bq m^−3^.

Secondly, according to Greaves (2002) the initiating step in childhood leukaemia is believed to take place *in utero*. This raises the question of whether radon measurements in the home of childhood cancer cases post diagnosis is the appropriate metric to employ. Kohli *et al* (2000) suggest that assessment of general radon exposure in the neighbourhood surrounding the home may be a better measure of the average exposure of the child, taking account of the time spent away from home, for example at school or play centres. This may also be a more appropriate metric for the exposure of the mother during pregnancy and of the transplacental transfer of radon and its decay products to the fetus. Kohli *et al* (2000) used measurements of radon in the ground to assess overall indoor radon exposure of childhood cancer cases in Sweden. The authors found evidence that children born and continuously living in areas with normal to high levels of radon have a significantly higher risk of childhood malignancy.

Thirdly, a number of studies have indicated an association between either childhood leukaemia or childhood cancer generally and urban air pollution, especially from motor vehicle exhausts (Savitz and Feingold, 1989; Knox and Gilman, 1997; Feychting *et al*, 1998; Harrison *et al*, 1999; Pearson *et al*, 2000). There are also studies suggesting an association between paternal exposure to hydrocarbons and increased leukaemia risk in their offspring (Savitz and Chen, 1990) and of similar exposures to mothers during pregnancy (Shu *et al*, 1999). Thus the effects of urban pollution may also act to confound radon measurement between cases and controls.

It may be that the UKCCS Investigators can address some of these issues in their existing data. However, the possibility may have to be faced that a link between radon and childhood cancer at the level suggested by radiation risk factors, as well as some geographical studies, is undetectable in a case–control design.

The impact of this is to recognise that while radon is not a major factor for childhood leukaemia or childhood cancer generally in the UK, it may be so in countries with much higher indoor radon levels, notably in Scandinavia. Also, it must be noted that the absence of an association in the UK study does not equate to the absence of an effect.
